# Supplementary material for: Development and validation of an automated machine for self-injury assessment via young Koreans’ natural writings
Source: PLoS One. 2025 Jan 16;20(1):e0316619. doi: 10.1371/journal.pone.0316619 (PMC11737660; doi:10.1371/journal.pone.0316619)
Supplement: S1 Table — Key = key tokens; rep. score = tokens’ representative scores; prop. est. = topic proportion estimates. (DOCX) [file pone.0316619.s001.docx]

**Supporting information**

**S1 Table. K-SITR codebook example.**

| **sub**  **set** | **key** | **rep. score** | **topic** | **title** | **prop. est.** |
| --- | --- | --- | --- | --- | --- |
| B | Parents | 145 | 6 | Self-stigma | 237.5808 |
| C | Parents | 117 | 8 | Familial conflicts - academic grades | 139.4267 |
| B | Depression | 113 | 5 | Adolescence psychopathology | 463.5253 |
| C | Depression | 108 | 5 | Academic stress | 242.543 |
| C | Middle school | 90 | 2 | Familial conflicts - adolescence career 1 | 138.7707 |
| B | Middle school | 77 | 5 | Adolescence psychopathology | 463.5253 |
| D | Parents | 50 | 3 | Difficulties in communicating at home | 109.633 |
| A | Depression | 49 | 3 | Adolescence psychological issues | 203.9831 |
| C | High School | 45 | 1 | Interpersonal issues | 134.4798 |
| A | Middle school | 44 | 3 | Adolescence psychological issues | 203.9831 |
| A | Parents | 41 | 8 | Lack of familial support | 101.2873 |
| B | High School | 40 | 5 | Adolescence psychopathology | 463.5253 |
| C | Stress | 39 | 5 | Academic stress | 242.543 |
| D | Depression | 39 | 4 | Adolescence mood issues | 145.7196 |
| B | Teacher | 34 | 4 | Self-injury provoking vulnerability factors | 178.1953 |
| C | Elementary school | 33 | 6 | Familial conflicts - parent-child, siblings | 76.3173 |
| D | Middle school | 29 | 4 | Adolescence mood issues | 145.7196 |
| C | Story | 27 | 1 | Interpersonal issues | 134.4798 |
| B | Stress | 26 | 3 | Unstable self | 121.2575 |
| B | Psychiatric hospital | 24 | 5 | Adolescence psychopathology | 463.5253 |
| A | Stress | 24 | 3 | Adolescence psychological issues | 203.9831 |
| C | Teacher | 23 | 5 | Academic stress | 242.543 |
| B | Elementary school | 22 | 4 | Self-injury provoking vulnerability factors | 178.1953 |
| A | Teacher | 21 | 1 | Adolescence school maladjustment 1 | 37.01894 |
| B | Story | 20 | 5 | Adolescence psychopathology | 463.5253 |
| C | Self-esteem | 19 | 1 | Interpersonal issues | 134.4798 |
| B | Psychiatric hospital | 18 | 4 | Self-injury provoking vulnerability factors | 178.1953 |
| C | Puberty | 18 | 1 | Interpersonal issues | 134.4798 |
| C | Psychiatric hospital | 18 | 4 | Interest in psychological treatment | 40.43559 |
| C | Girl | 16 | 5 | Academic stress | 242.543 |
| C | Grandmother | 16 | 2 | Familial conflicts - adolescence career 1 | 138.7707 |
| C | Psychological disorder | 15 | 3 | Familial conflicts - adolescence career 2 | 75.0916 |
| D | Teacher | 15 | 7 | Lack of surrounding support | 39.68774 |
| B | Grandmother | 14 | 6 | Self-stigma | 237.5808 |
| A | Elementary school | 14 | 8 | Lack of familial support | 101.2873 |
| D | Stress | 13 | 4 | Adolescence mood issues | 145.7196 |
| D | Story | 13 | 3 | Difficulties in communicating at home | 109.633 |
| A | Girl | 13 | 9 | Peer relationship struggles | 38.825 |
| B | Girl | 12 | 5 | Adolescence psychopathology | 463.5253 |
| C | Covid-19 | 12 | 5 | Academic stress | 242.543 |
| A | High School | 12 | 3 | Adolescence psychological issues | 203.9831 |
| B | Middle school | 12 | 4 | Self-injury provoking vulnerability factors | 178.1953 |
| B | Older man | 12 | 4 | Self-injury provoking vulnerability factors | 178.1953 |
| D | Elementary school | 12 | 3 | Difficulties in communicating at home | 109.633 |
| A | Stepmother | 12 | 6 | Familial conflicts 1 | 33.56167 |
| B | Elementary school | 11 | 4 | Self-injury provoking vulnerability factors | 178.1953 |
| B | Puberty | 11 | 4 | Self-injury provoking vulnerability factors | 178.1953 |
| B | Bipolar disorder | 10 | 5 | Adolescence psychopathology | 463.5253 |

Key = key tokens; rep. score = tokens’ representative scores; prop. est. = topic proportion estimates.
