# Supplementary figures and images for: Development and validation of an automated machine for self-injury assessment via young Koreans’ natural writings
Source: PLoS One. 2025 Jan 16;20(1):e0316619. doi: 10.1371/journal.pone.0316619 (PMC11737660; doi:10.1371/journal.pone.0316619)

**Supporting information**

**S1 File. K-SITR client report example.**


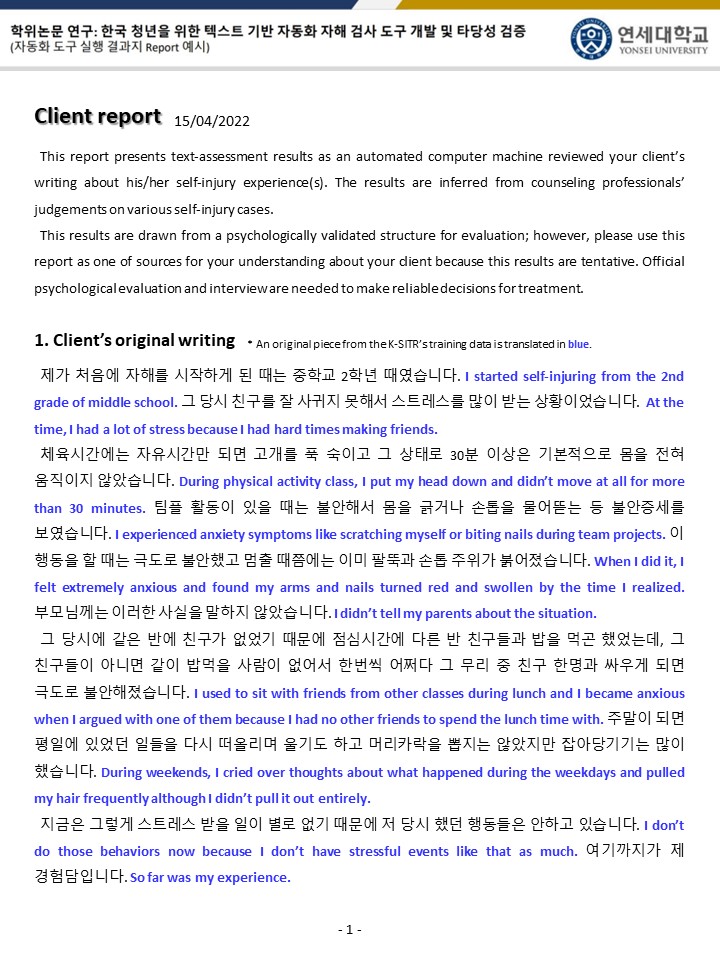


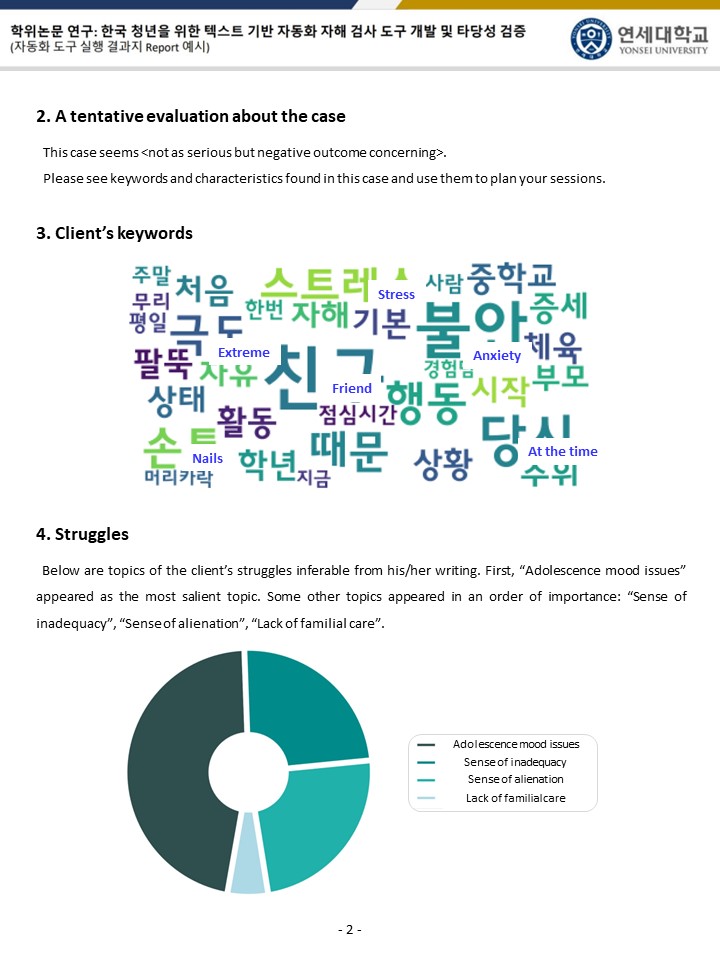


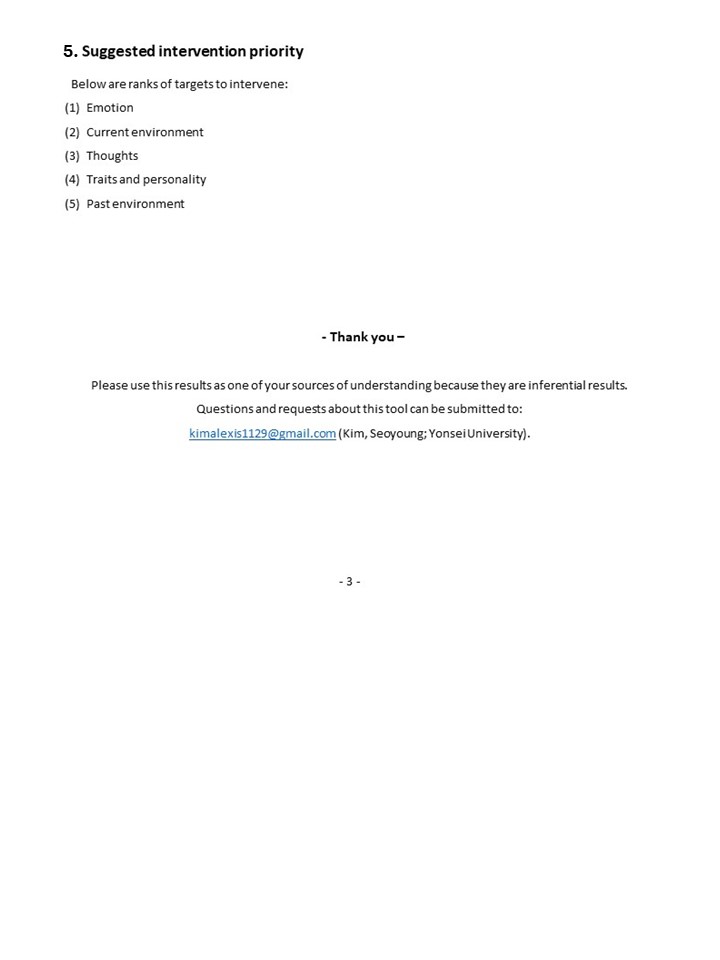

Supplement: S1 File — (DOCX) [file pone.0316619.s002.docx]
